# Quantum optical immunoassay: upconversion nanoparticle-based neutralizing assay for COVID-19

**DOI:** 10.1038/s41598-021-03978-2

**Published:** 2022-01-24

**Authors:** Navid Rajil, Shahriar Esmaeili, Benjamin W. Neuman, Reed Nessler, Hung-Jen Wu, Zhenhuan Yi, Robert W. Brick, Alexei V. Sokolov, Philip R. Hemmer, Marlan O. Scully

**Affiliations:** 1 Institute for Quantum Science and Engineering, Texas A&M University, College Station, TX 77843 USA; 2 Department of Biology, Texas A&M University, College Station, TX 77843 USA; 3 Global Health Research Complex, Texas A&M University, College Station, TX 77843 USA; 4 Department of Chemical Engineering, Texas A&M University, College Station, TX 77843 USA; 5 Baylor University, Waco, TX 76798 USA; 6 Department of Electrical and Computer Engineering, Texas A&M University, College Station, TX 77843 USA; 7 Zavoisky Physical-Technical Institute, Federal Research Center “Kazan Scientific Center of RAS”, Sibirsky Tract, Kazan, Russia 420029

**Keywords:** Nanoparticles, Nanoscale biophysics, Biological physics, Optical sensors

## Abstract

In a viral pandemic, a few important tests are required for successful containment of the virus and reduction in severity of the infection. Among those tests, a test for the neutralizing ability of an antibody is crucial for assessment of population immunity gained through vaccination, and to test therapeutic value of antibodies made to counter the infections. Here, we report a sensitive technique to detect the relative neutralizing strength of various antibodies against the SARS-CoV-2 virus. We used bright, photostable, background-free, fluorescent upconversion nanoparticles conjugated with SARS-CoV-2 receptor binding domain as a phantom virion. A glass bottom plate coated with angiotensin-converting enzyme 2 (ACE-2) protein imitates the target cells. When no neutralizing IgG antibody was present in the sample, the particles would bind to the ACE-2 with high affinity. In contrast, a neutralizing antibody can prevent particle attachment to the ACE-2-coated substrate. A prototype system consisting of a custom-made confocal microscope was used to quantify particle attachment to the substrate. The sensitivity of this assay can reach 4.0 ng/ml and the dynamic range is from 1.0 ng/ml to 3.2 $\upmu$g/ml. This is to be compared to 19 ng/ml sensitivity of commercially available kits.

## Introduction

The COVID-19 pandemic has shown how researchers equipped with the proper tools can rapidly translate scientific advances into improvements in healthcare as in the case of rapid viral genome sequencing^1^, proliferation of rapid antigen^2,3^, antibody^4–9^ and nucleic acid tests^10,11^, rapid determination of new protein structures^12^, and vaccines based on a stabilized version of the viral spike protein^13^. In such a pandemic, vaccination is anticipated to be the main tool to control the rapid spread of infections, and subsequent hospitalizations. Ideally, this paradigm works at its best when every vaccinated individual produces antibodies of sufficient strength and specificity to neutralize the virus. In practice, variations among individuals, population dynamics of antibodies, and frequent mutations of the virus can quickly reduce the effectiveness of vaccines^14–16^. One of the pandemic management tools still lacking improvement is a quick, reliable assay to measure the presence of neutralizing antibodies in serum. This would facilitate decisions on the timing of revaccination and calculations of herd immunity, and would provide a probe for the ever-growing pool of viral variants^17^. In addition, laboratory-generated antibodies, produced as antibody therapeutic treatments, need to be evaluated with a sensitive test to quantify their neutralizing potential.

The neutralizing antibody is defined by its ability to prevent the virus from interacting with a susceptible cell in a way that leads to infection^18–20^. Neutralizing antibodies first appear about two weeks after vaccination^21^, at roughly the same time that protection becomes evident^22^. Current methods to measure neutralizing level of antibodies require live cells and either intact SARS-CoV-2^23^ or virus-like particles consisting of a generic shell decorated with SARS-CoV-2 spike proteins^24^, and are prohibitive in terms of cost, expertise, and time for wider point of care use.

In the recent years, the fluorescent detection of single biomolecule has gained popularity^25–30^. However, photobleaching of the fluorescent dyes remains challenging for fluorescent readout. In addition, high-resolution microscopy and a longer acquisition time are often needed for single molecule detection, limiting its applications^24–30^. To resolve the photobleaching issue, the use of lanthanide doped upconversion nanoparticles (UCNPs) as fluorescent tags has been proven to be beneficial. For instance, Farka et al. use UCNPs as their fluorescent tags to detect prostate-specific antigen with a sensitivity of 1.2 pg/ml (42 fM) in 25% serum^31^. UCNPs can be excited by infrared lasers and remain stable after long exposure. In addition, the use of a high-power laser in a wide-field illumination configuration can increase the fluorescent signals and reduce the acquisition time, leading to faster measurements. It has been shown that using highly stable UCNPs could improve the limit of detection (LOD) of upconversion-linked immunosorbent assay (ULISA) by an order of magnitude compared to commercially available assays^30,31^. More sensitive optical readout can also improve the LOD in such bioassays^32,33^. These progressions toward single molecule detection are pushing the sensitivity, specificity, and LOD beyond what was once theoretically possible^34–36^.

Here, we show a proof of concept for a safe, simple, low-cost assay to determine the neutralizing activity of anti-SARS-CoV-2 antibodies using the tools of quantum optics. We use fluorescent UCNPs to measure the relative effectiveness of antibodies in preventing the binding of SARS-CoV-2 receptor-binding domain (RBD) to angiotensin-converting enzyme 2 (ACE-2). The method proposed in this study is in keeping with the principle that SARS-CoV-2 neutralizing antibodies could prevent the interaction between the RBD of the viral spike protein with the ectodomain of ACE-2^37^. For one clone of antibody used, we calculated the midpoint inflection point (IC50) to be 12 ng/ml (80 pM) and the LOD, defined as a concentration two standard deviations lower than the mean negative control value, to be 4 ng/ml (33 pM).

## Results and discussion

### The upconversion nanoparticle-based Neutralizing Immunoassay Kit (UNIK)

**Figure 1:**
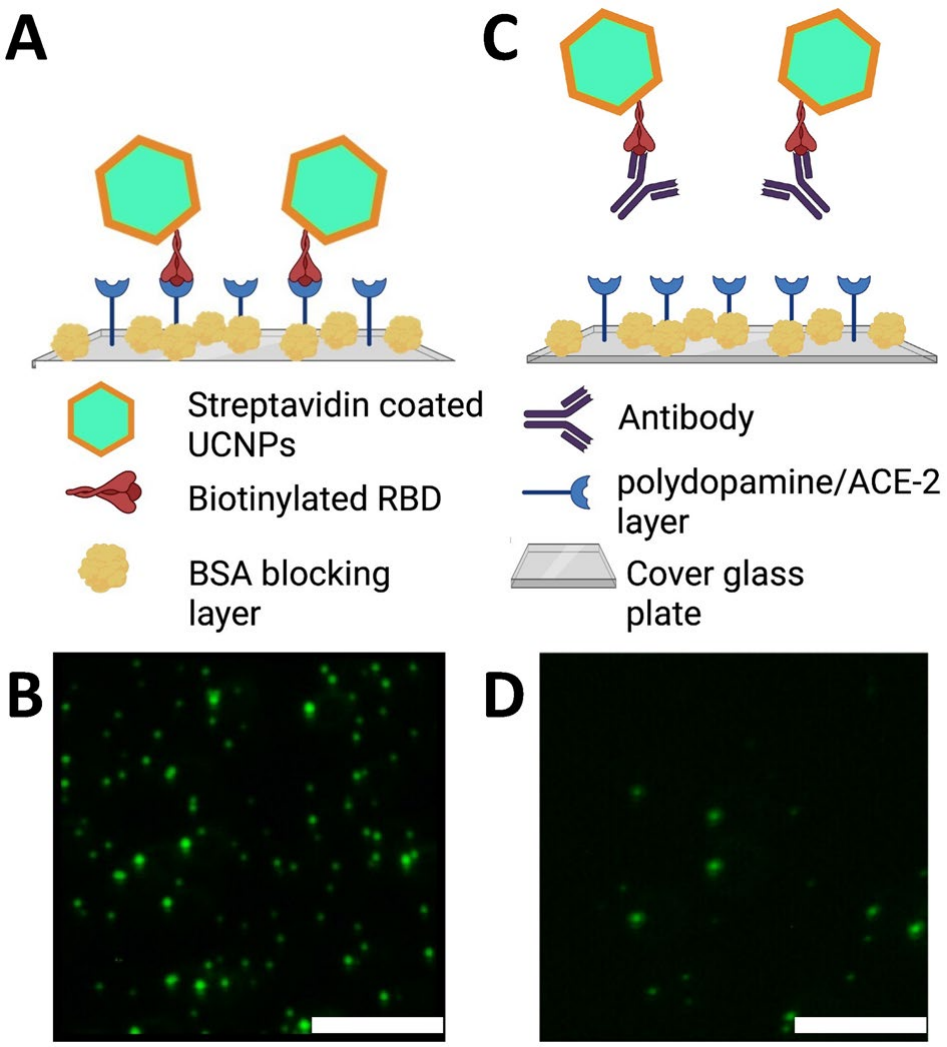
Schematic illustration of the upconversion-based neutralizing assay. (**A**,**B**) When the antibody is not present (or it is not neutralizing), the phantom virion complex will bind to the ACE-2-coated substrate and particles can be imaged and counted as shown in (**B**). The concentration of UCPVs was 0.4 $\upmu$g/ml and no antibodies were present in the solution. (**C**,**D**) When the antibody is present and it is neutralizing, it will prevent the phantom virion complex from binding to ACE-2-coated substrate and as a result, fewer fluorescent particles will be observed compared to the negative control as shown in (**D**). The concentration of UCPVs was 0.4 $\upmu$g/ml and the concentration of the antibody was 3.23 $\upmu$g/ml. Scale bars represent 15 $\upmu$m. (**A**,**C**) Were created with BioRender.com.

The basic principle of the upconversion nanoparticle-based neutralizing immunoassay kit (UNIK) is shown in Fig. 1. The assay relies on the natural affinity between RBD and ACE-2 protein. To fully take advantage of this property, we employed streptavidin conjugated upconversion nanoparticles and biotinylated RBD to produce the upconversion nanoparticle phantom virion (UCPV). If there is no antibody present in the sample (or if the antibodies present in the sample are non-neutralizing), the phantom virion particles will bind to the substrate without any obstruction. As a result, images taken from these samples will show high count of particles (Fig. 1A,B). On the other hand, if the antibody is effectively neutralizing the RBD, then the binding of phantom virion and ACE-2 will be hindered, thus a lower count of particles will be observed in the images (Fig. 1C,D), compared to the negative control sample with no antibody present, as shown in Fig. 1A,B.

**Figure 2:**
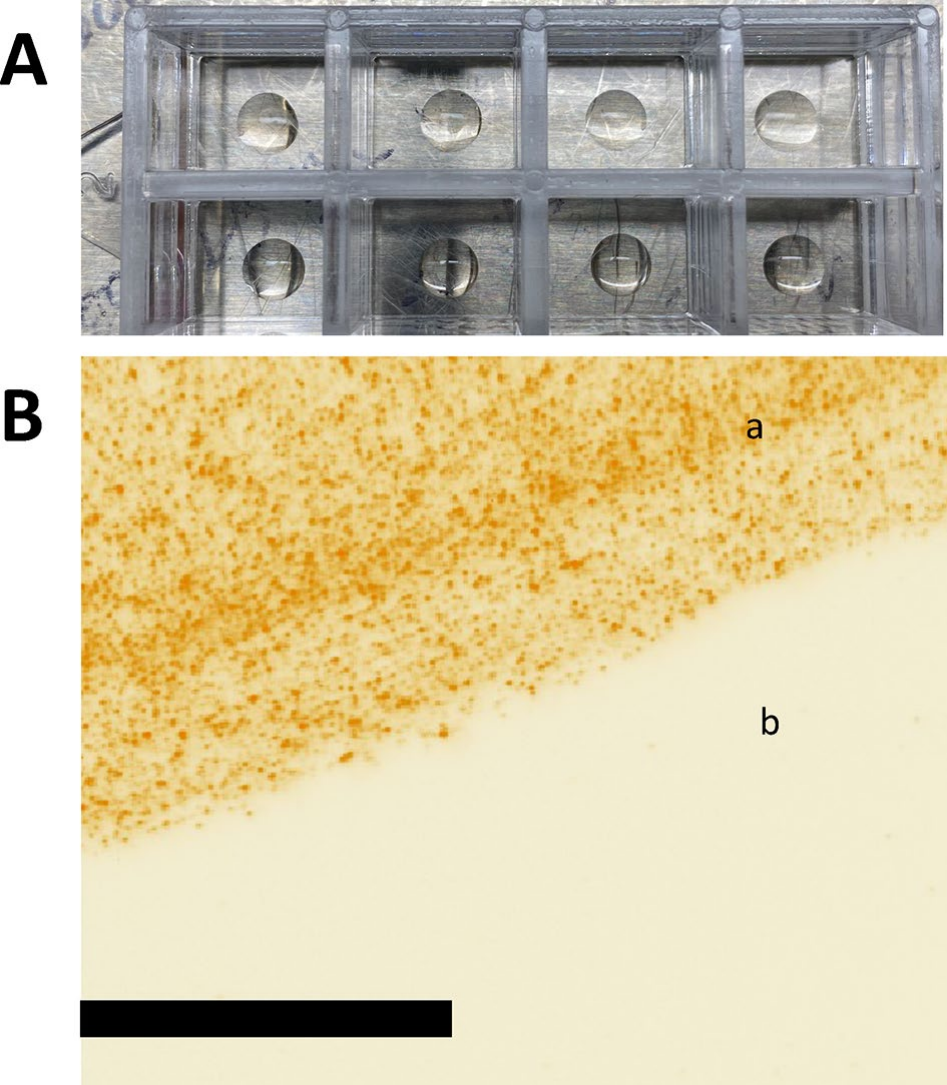
Affinity of UCPV and ACE-2 coated area. (**A**) Typical configuration of the coated area on the Nunc LabTek II 8-well dishes with bottom cover glass. The volume of the ACE-2 coating was (10 $\upmu$l). For the rest of the steps in all experiments, the whole well was filled (as described in methods and supplementary materials). (**B**) After polydopamine/ACE-2 coating and blocking, we incubated the plate with UCPV solution (10 $\upmu$g/ml). The image was taken from the edge of the coated area. The area coated with ACE-2 (a in **B**) shows a high fluorescence particle count, while the uncoated and BSA blocked area shows no particles at all (b in **B**). This shows a relatively very minimal nonspecific binding between the blocked cover glass and UCPVs.

**Figure 3:**
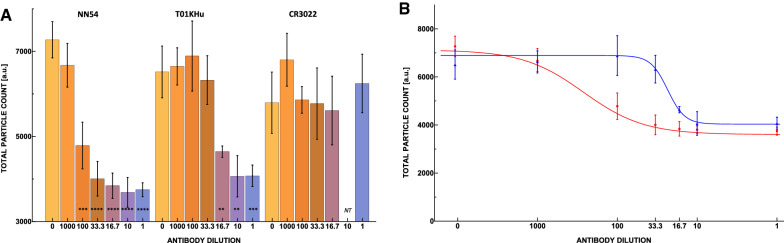
(**A**) Neutralizing activity of neutralizing antibody clones NN54 (left) and T01KHu (middle), against non-neutralizing but binding antibody clone CR3022 (right), presented as total particle count of 10 images averaged over 3 repetitions for each reciprocal dilution factor. The highest concentration was 3.23 $\upmu$g/ml. Each bar is tagged with the equivalent *p* value star designation. NT means not tested. (**B**) 4-parameter logistic curve fitted to neutralizing antibodies NN54 (red) and T01KHu (blue) data. IC50 for NN54 and T01KHu were 12 ng/ml (80 pM, 1:269 dilution factor) and 138 ng/ml (933 pM, 1:23 dilution faction), respectively. The Hill coefficients for NN54 and T01KHu were calculated to be 1.148 and 4.0836, respectively, as described in “IC50 and Hill coefficient”.

**Figure 4:**
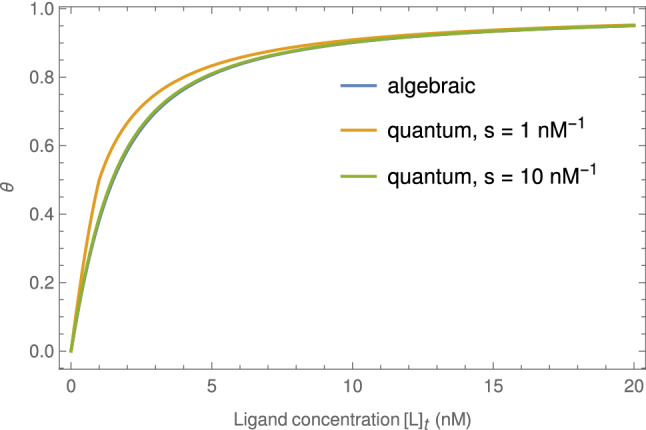
Comparison of $\theta$, the concentration ratio of protein–ligand complexes to total protein, computed by 2 and 10 for the case of $K_d=[P]_t=1\text { nM}$.

**Table 1:** Antibody concentrations and volumes used to prepare each final concentration.

Final antibody concentration (in $\upmu$g/ml)	UCPV stock solution (0.4 $\upmu$g/ml) volume used (in $\upmu$l)	Antibody stock solution concentration (in $\upmu$g/ml)	Dilution ratio	Antibody stock solution volume used (in $\upmu$l)
0.00323	300	0.1	1:1000	10
0.0323	300	1	1:100	10
0.0968	300	3	1:33.3	10
0.194	300	6	1:16.7	10
0.323	300	10	1:10	10
3.23	300	100	1:1	10

### Assessment of ACE-2/polydopamine coated plates

Glass bottom plates were coated with ACE-2/polydopamine mixtures. The activity of ACE-2 was evaluated by measuring the binding between ACE-2 and SARS-CoV-2 RBD that is linked to mouse IgG Fc tag. RBD was further detected by goat anti-mouse antibody with Alexa fluor 633. The fluorescent spectra are shown in Supplementary Figure S4 (B, D, E, H). To make sure that nonspecific bindings or autofluorescence signals were minimal, several control experiments were performed (Supplementary Figure S4). Supplementary Figure S4A shows the positive control test, in which the plates were coated with ACE-2/polydopamine mixture and then blocked with 5% BSA solution (Supplementary Materials). The next layer was RBD with mouse Fc tag which was detected with goat anti-mouse antibody with Alexa fluor 633. Supplementary Figure S4B shows the spectrum of the Alexa fluor obtained with a 638 nm laser. This spectrum clearly shows the positivity of this sample. Supplementary Figure S4C shows the negative control test, in which instead of RBD with Fc tag, the plate was incubated 1$\times$ PBS as a negative control sample. Supplementary Figure S4D shows the spectrum of the Alexa fluor obtained with 638 nm laser from this sample. This spectrum clearly shows the negativity of this sample, as there is only background readout signal. Supplementary Figure S4E shows the control sample which is missing the goat anti-mouse antibody with Alexa fluor 633. Since in these measurements the excitation laser was the 638 nm laser, there was a possibility of autofluorescent background from any of the elements on the plate. To check if there was any autofluorescence, we prepared this sample and scanned it. Supplementary Figure S4F shows the spectrum of the Alexa fluor obtained with the 638 nm laser from sample shown in Supplementary Figure S4E. This spectrum clearly shows the negativity of this sample, as there is only background readout signal. So, there are minimal autofluorescent signals from other elements on the plate. Supplementary Figure S4G shows the control test which is missing the ACE-2 protein. This plate was coated with mixture of 1$\times$ PBS and polydopamine and then blocked with 5% BSA. The purpose of this test was to measure the extent of nonspecific binding of RBD with mouse Fc tag and secondary Alexa fluor conjugated antibody complexes with ACE-2-coated plates. Supplementary Figure S4H shows the spectrum of the Alexa fluor obtained with 638 nm laser from Supplementary Figure S4G. This spectrum shows a small background in this sample. However, the positive signal shown in Supplementary Figure S4B is approximately 16 times larger than this background.

### Nonspecific binding

One of the concerns in using any type of plate for bioassays is nonspecific binding. The nonspecific binding between the phantom virion and ACE-2-coated plates can increase the background signal, thus decrease the LOD and sensitivity. Polydopamine molecules, which are positively charged, can bind to the phantom virion without involvement of ACE-2 and RBD. In addition, any imperfection on the plate’s coating or UCPVs can increase the nonspecific binding. To assess this, we prepared ACE-2/polydopamine-coated plates and 1 × PBS/polydopamine coated plates, both blocked with 5% BSA to show that phantom virion particles only bind to substrate when ACE-2 protein is mixed with polydopamine and plated on the substrate. To test the affinity of the particles with the ACE-2 coated plates and compare it with blank and blocked plate (no antibody was used), we imaged the edge of the coated area (the location of the edge was found by rough marking on the glass plate and the coffee-ring effect of the coated area after it was dried). Figure 2A shows the 10 $\upmu$l coating of polydopamine/ACE-2 protein. Since only a certain area of each well was coated, we expected to find the particles only in that coated region. Figure 2B shows very high binding between phantom virion particles and the coated area, while the uncoated area to the side did not show any nonspecific binding between UCPV and the blocked blank glass coverslip (effective imaged area is 145 $\upmu$m by 145 $\upmu$m).

We also coated plates with a polydopamine and PBS mixture and blocked them with 5% BSA blocking buffer to study nonspecific bindings that may arise between polydopamine and UCPVs (Supplementary Figure S5). We expected to see a very low count of particles on these plates, based on this assumption that there is no affinity between UCPVs and polydopamine. Supplementary Figure S5 shows three images of three different areas of the same sample taken from the center of the coated area, and only a few of particles are visible in the images (effective imaged area is 145 $\upmu$m by 145 $\upmu$m). The particles appear as small diffraction-limited green spots on the dark background of the images. These results (Fig. 2 and Supplementary Materials Figures S4 and S5) prove that the binding between ACE-2-coated area and UCPV is specifically caused by the natural affinity between RBD and ACE-2 proteins. It is important to note that those nonspecific bindings are due to surface imperfections of the substrate and the particles, as well as the protein coating integrity of the particles and substrate. For instance, excessive sonication (which is a step of the UCPV preparation procedure; see supplementary materials) can damage and denature the protein coating of the nanoparticles, either due to excessive heating or high-pressure waves generated by bath sonicator inside the nanoparticle vials. Optimization of every step and paying attention to such details can decrease the amount of nonspecific binding.

### IC50 and Hill coefficient

To test the neutralizing ability of the antibodies using UCPVs, we made a serial dilution of the antibody clones NN54, T01KHu, and CR3022 and mixed equal volumes of the antibody dilutions with equal volume and concentrations of UCPVs (Table 1). The dilutions were calculated such that the final sample volume on each plate was the same for all samples as was the concentration of UCPVs. But the concentration of the antibodies was different in each sample (i.e., the ratio of particles to antibody was different for each sample). Table 1 shows the final antibody concentration for each data point.

According to the manufacturer’s datasheet for neutralizing antibody NN54, the ELISA-based neutralizing assay kit performed on this antibody showed an average IC50 point (defined below) of 0.857 nM (0.129 $\upmu$g/ml)^38^. As for neutralizing antibody T01KHu, the manufacturer reports the lowest IC50 point to be at 0.1 $\upmu$g/ml^39^. For CR3022, it has been reported that this antibody does not block binding of RBD with ACE-2 protein^40^.

IC50 point in the current work is defined as the concentration where the signal count is (maximum count – minimum count)/2 estimated by the fitting 4-parameter logistic function:

1 $$\begin{aligned} Y=A+\frac{B-A}{1+(Conc/IC50)^{hc}}, \end{aligned}$$

where *Y* is the total count, *A* is the minimum count, *B* is the maximum count, *Conc* in the concentration of antibody used, *IC*50 is the concentration of antibodies at which the count is at 50% and *hc* is the Hill coefficient (see supplementary materials for fitted functions).

The IC50 points are calculated to be 12 ng/ml (80 pM) and 138 ng/ml (933 pM) for NN54 and T01KHu (Fig. 3B), respectively. The assay is also capable of differentiating between antibodies’ respective Hill coefficients in the context of their interaction with UCPVs. The Hill coefficient has been used to measure the cooperativity of multivalent binding systems^41,42^. In the dose-response curves, the Hill coefficients for NN54 and T01KHu were calculated to be 1.148 and 4.0836, respectively. Comparing with NN54, the binding of T01KHu is closer to multiple ligand interactions. These parameters were calculated by fitting the 4-parameter logistic function to the data sets using an online tool (supplementary materials)^37^. Thus, we can differentiate between antibodies in terms of their strength (IC50) and cooperativity (Hill coefficients) in binding to the UCPVs.

Figure 3A,B show the effectiveness of neutralizing antibodies NN54 and T01KHu as their concentration increases. They also show that the non-neutralizing clone CR3022 does not prevent UCPVs from binding to ACE-2 coated plates. Our assay can differentiate the IC50 point with high sensitivity and determine the antibody with higher affinity without the use of enzymatic enhancement in ELISA. This assay also shows differences in the Hill coefficients for these two antibodies, which shows that T01KHu is a multiligand interaction while NN54 seems to be a single ligand interaction.

One should be cautious when comparing the results in Fig. 3 with reported results from other sources, such as ones reported by the manufacturers of the antibodies and tests. For instance, manufacturer of NN54 reports two IC50s for the same antibody from two different neutralizing tests. In one, they report an average IC50 of 1.41 $\upmu$g/ml obtained from neutralizing assays involving 293T/ACE-2 cells^38^. These cells were infected with Pseudotyped Luciferase rSARS-CoV-2 Spike and the concentration of neutralizing antibody was changed to see the how many cells were not infected by the spike^38^. In the other test, they report an IC50 of 0.129 $\upmu$g/ml measured using an inhibitor screening ELISA kit. The question of what the correct IC50 value is, seems irrelevant since the parameters of these tests are different, as are their goals. In short, each test is optimized for certain dynamic range and specific LOD.

To understand how test parameters interplay with LOD for instance, one can take note of the ratio of protein–ligand complexes and total protein molecules ($\theta$ value). One can assume, for simplicity, that ACE-2 is the protein and UCPV is the ligand in our test. The combination of enzymatic reaction and RBD in a neutralizing ELISA test is equivalent to the UCPV in our test. The ratio of protein–ligand complexes to the total proteins, $\theta$, is (see supplementary materials for proof):

2 $$\begin{aligned} \theta =\frac{[PL]}{[P]_{t}}=\frac{([P]_{t}+[L]_{t}+[K]_{d})-\sqrt{([P]_{t}+[L]_{t}+[K]_{d})^2-4[P]_{t}[L]_{t}}}{2[P]_{t}}, \end{aligned}$$

where $\theta$ is the ratio of protein molecules bound to ligand. $[PL],[P]_{t}, [L]_{t}$, and $K_{d}$ are total concentration of protein–ligand complex, total concentration of protein, total concentration of ligand, and the dissociation constant of protein and ligand respectively. The concentration of $[L]_{t}$ when $\theta =0.5$ (IC50 concentration) can be derived from Eq. (2) with simple algebra as

3 $$\begin{aligned} \left. {[}L]_{t}\right| _{\text {IC50}}=\frac{1}{2}[P]_{t}+K_{d}. \end{aligned}$$

As can be seen, the IC50 concentration in reality depends on two parameters. One is the total protein concentration and the other is the $K_{d}$ value. Using Eq. 3, for the more complex case of our assay, we can derive the following relation for the antibody concentration (see supplementary materials for proof)

4 $$\begin{aligned} \left. {[}Ab]\right| _{\text {IC50}}=[UCPV]_{total}-K_{d}^{(2)}+\frac{2[UCPV]_{total}K_{d}^{(2)}}{[ACE]+2K_{d}^{(1)}}-\frac{1}{2}[ACE]-K_{d}^{(1)} \end{aligned}$$

where $[UCPV]_{total}$ is the total UCNP concentration, [ACE] is the total ACE-2 protein concentration, $K_{d}^{(1)}$ is the dissociation constant between UCPV and ACE-2, and $K_{d}^{(2)}$ is the dissociation constant between UCPV and neutralizing antibody. In our work, only the UCPV concentration and ACE-2 concentration can be controlled and manipulated to reduce the IC50 concentration. Supplementary Figure S7 shows the changes in $\left. [Ab]\right| _{\text {IC50}}$ as a function of ACE-2 concentration, for different values of UCPV concentrations. As can be seen, we needed to maximize the amount of ACE-2 protein on the substrate, while optimizing the UCPV concentration to the lowest amount possible. Rationally, by decreasing UCPVs, we reduced the number of antibodies needed to fully block them, while by maximizing ACE-2 protein we increased the number of unblocked UCPVs captured on the substrate.

In the case of a test such as ELISA, reducing the RBD concentration means fewer actual RBD–ACE complexes will be available to be detected later on (through anti-ligand secondary antibodies and enzymatic enhancement, fluorescent dyes, etc). Since the LOD is an arbitrary choice, and we can choose IC50 concentration for this simple examples, we can conclude that different tests involve different amounts of protein concentrations and are optimized for specific dynamic ranges and different LOD.

A keen observer may ask: for NN54 and T01KHu antibodies, why do manufacturers report similar IC50s concentrations of 0.129 $\upmu$g/ml and 0.1 $\upmu$g/ml respectively. Supplementary Figure S8 illustrates the reason for similar results from companies. When we set $[P]_{t}=19\text { nM}$, $K_{d}=1\text { nM}$ and $[P]_{t}=1\text { nM}$, $K_{d}=10\text { nM}$, we see that both cases have the same IC50 concentrations where clearly, we assumed different $K_{d}$ values. The difference in the conditions of the manufacturer’s test is perhaps the reason for their similar results.

The advantage of UNIK is apparent from two important factors. First, it can differentiate between IC50 concentrations and Hill coefficients of two different neutralizing antibodies. Second, although the limit of detection (LOD, defined Sect. 2.8) is a function of both UCPV concentration and the affinity between antibody and UCPV, and affinity between UCPV and ACE-2 (the corresponding $K_{d}$ values, as shown in Eq. 4), its LOD is an order of magnitude better than that of cited commercial tests, among which the best LOD is reported to be 19 ng/ml^43^. Our results show that proper optimization of UCPV’s concentration, while maximizing the number of RBD per UCNP (120:1) and maximizing ACE-2 protein on the substrate can improve LOD. This is because we can detect single molecule bindings and as such we can reduce UCPV concentration to such low amounts that a lower concentration of neutralizing antibody will be needed to block them while UCPVs can still be detected (described in Eq. 4). The dependence of LOD to $K_{d}$ value was also shown by S. Zhang et al.^36^. As depicted by Eq. 4, factors that play a role in UNIK’s LOD are $K_{d}$ value between UCPV and ACE-2 protein, $K_{d}$ value between the UCPV and antibody, total concentration of UCPV, and total concentration of ACE-2 protein. It is best to derive such equation for every specific assay to maximize the improvement in the LOD.

### Quantum description of $\theta$

It is possible to give a “quantum” (i.e., discretized and probabilistic) alternative to the previous section’s purely algebraic treatment of the protein–ligand binding process $P+L\rightleftharpoons PL$. The basic idea is to view the population *n* of the protein–ligand complex *PL* as a random variable that can take the discrete values $n=0,1,\ldots$. Its probability distribution $\{p_n\}$ evolves in time toward an equilibrium distribution, due to the competing reactions of birth (unit increase in *n* when one *P* and one *L* bind together to form *PL*) and death (unit decrease in *n* when one *PL* dissociates). Such a model is known in probability theory as a birth–death process^44^. The birth–death formalism has found numerous scientific applications, notably in obtaining the photon statistics in the quantum theory of the laser^45^.

In more detail, we posit a birth rate from state *n* to $n+1$ of $\lambda _{n}=\alpha (a-n)(b-n)$ and a death rate from *n* to $n-1$ of $\mu _{n}=\beta n$. Here $\alpha >0$ and $\beta >0$ play the role of binding and dissociation rate constants, while *a* and *b* denote the initial populations of *P* and *L*, which like *n* are constrained to be integers, but unlike *n* are specified precisely (not probabilistic). Then the probabilities $p_n$ are governed by the differential equations

5 $$\begin{aligned} \begin{aligned} \frac{dp_{0}}{dt}&=-\lambda _{0} p_{0}+\mu _{1} p_{1} \\ \frac{dp_{n}}{dt}&=-\lambda _{n} p_{n}+\lambda _{n-1} p_{n-1}-\mu _{n} p_{n}+\mu _{n+1} p_{n+1}&\text {when}~ n\ge 1. \end{aligned} \end{aligned}$$

We are interested in equilibrium behavior, so we seek the steady-state solution of 5, i.e. the probability distribution given by $\frac{dp_{n}}{dt}=0$, which is

6 $$\begin{aligned} p_{n}=p_{0} \prod _{k=1}^{n} \lambda _{k-1}/\mu _{k} \end{aligned}$$

with $p_{0}$ determined by the normalization $\sum _{n} p_{n} = 1$. For the given birth and death rates $\lambda _{n}$ and $\mu _{n}$, 6 can be described by the probability-generating function

7 $$\begin{aligned} G(z)=\sum _{n=0}^{{\text {min}}(a,b)} p_{n} z^{n}=\frac{_{2}F_{0}(-a,-b;;\frac{\alpha }{\beta }z)}{_{2}F_{0}(-a,-b;;\frac{\alpha }{\beta })} \end{aligned}$$

where $_{2}F_{0}$ is a generalized hypergeometric function. The expected population of *PL* at equilibrium is

8 $$\begin{aligned} \langle n\rangle _\text {eq}=G'(1)=ab \frac{\alpha }{\beta }\frac{_{2}F_{0}(1-a,1-b;;\frac{\alpha }{\beta })}{_{2}F_{0}(-a,-b;;\frac{\alpha }{\beta })}. \end{aligned}$$

With numerical computations in mind, it is desirable to cast 8 into a form better behaved with regard to continuity in its parameters. To that end we use an identity of hypergeometric functions^46^, Eq. 16.2.3] to get

9 $$\begin{aligned} \langle n\rangle _\text {eq}={\left\{ \begin{array}{ll}a\frac{_1F_1(1-a;1-a+b;-\frac{\beta }{\alpha })}{_1F_1(-a;1-a+b;-\frac{\beta }{\alpha })}&{}\text {if}~ a\le b\\ b\frac{_1F_1(1-b;1-b+a;-\frac{\beta }{\alpha })}{_1F_1(-b;1-b+a;-\frac{\beta }{\alpha })}&{}\text {if}~a\ge b \end{array}\right. } \end{aligned}$$

where $_1F_1$ is Kummer’s confluent hypergeometric function. With the aid of another identity^46^, Eq. 13.2.39] we finally have

10 $$\begin{aligned} \theta =\frac{1}{a}\langle n\rangle _\text {eq}=\frac{1}{a}\min (a,b)\frac{_1F_1(\max (a,b);1+|a-b|;\frac{\beta }{\alpha })}{_1F_1(1+\max (a,b);1+|a-b|;\frac{\beta }{\alpha })}. \end{aligned}$$

When evaluated numerically this expression agrees closely with the non-probabilistic treatment of the law of mass action 2 (see Fig. 4). To make the comparison precise, one should make the substitutions $a\rightarrow s [P]_t$, $b\rightarrow s[L]_t$, and $\beta /\alpha \rightarrow s K_d$ in 10, where *s* is a constant that converts units of concentration into number of molecules. In principle *s* is the product of the Avogadro constant and the experimental volume, though in practice the agreement between the $\theta$’s is already satisfactory for small values of *s*.

The antibody concentration at IC50, $\left. [Ab]\right| _\text {IC50}$, predicted by the quantum approach can be obtained by imitating the calculation in the previous section but using 10 instead of 2 to express equilibrium protein–ligand concentration. The result is indistinguishable from the algebraic result (see figures in the supplementary materials). In practice $\left. [Ab]\right| _\text {IC50}$ must be found using a numerical root-finding routine when employing 10, as the equation $\theta _1=1/2$ is not amenable to closed-form solution.

### UCPVs concentration optimization

In neutralizing assays such as UNIK, the antigen concentration plays an important role. In this work, RBD is the antigen and it is pre-bound to the UCNP. The concentration of RBD in the assay depends on two factors, number of RBD bound to each UCNP and final working concentration of UCPV. There are several method of optimization of UCNP–protein conjugate reported in the literature^30,31^. We decided to fix the number of RBD on the UCNP at 120 RBD per 1 UCNP and optimize the concentration of the resulting UCPVs^31^. Figure S3 shows the result of concentration optimization for our experiment. In the region between 0.1 $\upmu$g/ml and 1 $\upmu$g/ml the average number of particles counted per image changes rapidly, increasing with concentration of UCPVs. In this region, for this ratio of RBD to UCNP that we used, we have the maximum sensitivity. A slight decrease in the UCPV concentration due to presence of neutralizing antibodies will cause measurable changes in the countable particles in the images.

### Non-neutralizing antibody

It is important to differentiate an antibody that binds but does not neutralize infectivity from a truly neutralizing antibody, which would provide direct protection against infections. In the case of clone CR3022, its binding does not block the binding site on the RBD specific for the ACE-2 protein^40^. In a separate experiment, serial dilutions of CR3022 IgG antibody were mixed with UCPVs and tested (see descriptions in the supplementary materials). The results, illustrated in Fig. 3A, show that the average particle counts per scan in all these dilutions stay relatively close to the control sample (no statistical significance, *p* values$\ > 0.1$, see supplementary materials). In this work, we define neutralizing activity as binding of the antibody to RBD at the location where ACE-2 protein would bind. As such, CR3022 is not a neutralizing antibody^40^.

### Detection limit

The limit of detection (LOD) for this assay is defined as the concentration with a count of two times the negative control’s standard deviation below negative control’s average count^43^. Using this definition and the calibration curves, we estimated the LOD of this assay for both neutralizing antibodies, see supplementary materials for details. The LOD for NN54 and T01KHu are estimated to be 0.004 $\upmu$g/ml and 0.128 $\upmu$g/ml respectively. In addition, based on the *p* values of each data point, we can conclude that there is a statistical significance between 0.00323 $\upmu$g/ml (*p* value 0.19) and 0.0323 $\upmu$g/ml (*p* value 0.0034). Thus, in practice the detection limit for NN54 can be assumed to be 0.0323 $\upmu$g/ml. For the case of T01KHu, the statistical significance is first observed between 0.0968 $\upmu$g/ml (*p* value 0.71) and 0.196 $\upmu$g/ml (*p* value 0.0065) and as a result, the detection limit for this antibody can be assumed to be 0.196 $\upmu$g/ml.

### Assay modifiability

Other variations of this assay are also possible, adjusted for SARS-CoV-2 variants or other viral species. To modify this assay, one can place the RBD of other SARS-CoV-2 variants on the particles and ACE-2 protein on the substrate. It is also possible to use multiple upconversion nanoparticle types with different fluorescent emissions for each RBD variant. For instance, we can conjugate NaYF$_4$:Yb/Tm particles (excitation peak at 980 nm, emission bands around 375 nm and 450 nm) with the Alpha variant of SARS-CoV-2 RBD, and NaYF$_4$:Yb/Er particles (excitation peak at 980 nm, emission bands around 550 nm and 650 nm) with the Delta variant of SARS-CoV-2 RBD. Thus, we can test the antibodies against both variants of virus simultaneously. This is part of our future study plan.

## Conclusion

We have demonstrated that UNIK can be used effectively for determination of neutralizing activity of COVID-19 antibodies. We show that with proper optimization, we can detect the antibody for SARS-CoV-2 virus. By considering a protein–ligand binding model, we derived an expression for antibody concentration at 50% relative particle count and identified important parameters influencing the limit of detection. Although the limit of detection is dependent on the concentration of UCPV as well as the affinity of antibody and UCPV, as seen in the case of NN54 and T01KHu antibodies, we report that the lowest detection limit for this assay was 4 ng/ml (27 pM, calculated for neutralizing antibody clone NN54). A paper-based ELISA test for detection of COVID-19 antibodies reported 9.00 ng/$\upmu$l (i.e., 9.00 $\upmu$g/ml) limit of detection^47^. A readily available commercial neutralizing assay from Cayman Chemical reports a LOD of 19 ng/ml^43^. The assessment of the performance of the assay with blood serum samples as well as measuring the receiver operating characteristic curve (ROC curve) using human convalescent blood plasma, as well as other variation of the assay mentioned before are subjects of our future studies. We also showed that one must be cautious when defining the LOD, since both measured parameters depend on various factors, resulting in various LOD even under the same conditions but with different samples.

## Methods

All the incubation steps in this section were performed at room temperature unless mentioned otherwise.

Upconversion nanoparticle phantom virions (UCPV) were prepared as follows. Briefly, the streptavidin coated upconvertion nanoparticles were purchased from Creative Diagnostics (part numbers of all materials are listed in Supplementary Materials Table S1). The they were diluted to 200 $\upmu$l and concentration of 0.5 mg/ml and sonicated for 10 minutes. Then 10 $\upmu$l of 0.2 mg/ml biotinylated RBD was added to it and left on the vortex mixer for 1 hour at lowest speed. Subsequently, particles were washed 3 times by centrifuging, replacing the supernatant with fresh assay buffer, and sonicating for 10 minutes (more specific details on sonication and particle wash are in the supplementary materials). Then, the UCPVs were diluted down to 0.4 $\upmu$g/ml at a volume of 4 ml, sterile filtered using 0.2 $\upmu$m cellulose acetate syringe filters, and kept at 4$^{\circ }$C until use. There are more details on how the concentration of UCVPs was selected in supplementary materials.

The optimization of UCPVs concentration was as follows. We prepared 4 different concentrations of UCPVs (0.1 $\upmu$g/ml, 0.4 $\upmu$g/ml, 1 $\upmu$g/ml, 10 $\upmu$g/ml) and plated them onto the blocked plates as described below. No antibody was used in this measurement. Subsequently we took 5 images of each concentration, counted the particles and averaged the number per image. The results are shown in Supplementary Figure S3.

To prepare the Nunc Lab-Tek II coverglass plates, we mixed 0.75 mg/ml ACE-2 protein with 2 mg/ml polydopamine solution at 1:1 ratio (more details in the supplementary materials) and plated the solution on the coverglass plate wells. The plates were incubated for 2 hours and kept inside a humidity chamber to prevent drying. The plates were then washed 4 times with assay buffer (1$\times$ PBS, 0.5% BSA, 0.1% tween-20) and blocked with 5% BSA solution (1$\times$ PBS, 5% BSA, 0.1% tween-20) for 1 hour. Then the plates where washed again 4 times with assay buffer and used immediately. There are more details on validation of plates explained in the supplementary materials.

To perform the upconversion nanoparticle-based neutralizing immunoassay, different concentrations of the antibodies were prepared (Table 1). Then, 10 $\upmu$l of each concentration was added to a separate 300 $\upmu$l of 0.4 $\upmu$g/ml UCVP solution and left on the mixer for 1 hour. Then, each of the UCPV and antibody mixes was added to a separate wells of a prepared Nunc Lab-Tek II 8-well plate and incubated for 1 hour. After incubation, the plates were washed 4 times with assay buffer and kept at 4$^{\circ }$C until measurements. This procedure was repeated 3 times for the 3 different antibodies.

To count the number of nanoparticles on the plates, 10 images of each well were obtained using a custom-made confocal microscope (details of the system in supplementary materials). Then, the particles, observed as bright spots in the images (Fig. 1B,D), were counted and recorded for each final concentration of each antibody (Table 1) using a custom-made program in Mathematica software. The counts of 10 images of each data point were averaged for the 3 repetitions for that antibody. More details on how the images were taken and processed can be found in supplementary materials.

## Supplementary Information

Supplementary Information.
